# Transcriptome Profile of Nicotinic Receptor-Linked Sensitization of Beta Amyloid Neurotoxicity

**DOI:** 10.1038/s41598-020-62726-0

**Published:** 2020-03-30

**Authors:** Komal Arora, Mahdi Belcaid, Megan J. Lantz, Ruth Taketa, Robert A. Nichols

**Affiliations:** 1 Department of Cell and Molecular Biology, John A. Burns School of Medicine, University of Hawai’i, Honolulu, HI United States; 2 Pacific Center for Emerging Infectious Diseases Research, John A. Burns School of Medicine, University of Hawai’i, Honolulu, HI United States; 3 Present Address: Department of Biology, Georgia State University, Atlanta, GA United States; 4 Present Address: Hawai’i Institute of Marine Biology, University of Hawai’i at Manoa, Honolulu, HI United States

**Keywords:** Molecular neuroscience, Alzheimer's disease

## Abstract

Understanding the specific gene changes underlying the prodromic stages of Alzheimer’s disease pathogenesis will aid the development of new, targeted therapeutic strategies for this neurodegenerative disorder. Here, we employed RNA-sequencing to analyze global differential gene expression in a defined model nerve cell line expressing α4β2 nicotinic receptors (nAChRs), high-affinity targets for beta amyloid (Aβ). The nAChR-expressing neuronal cells were treated with nanomolar Aβ_1–42_ to gain insights into the molecular mechanisms underlying Aβ-induced neurotoxicity in the presence of this sensitizing target receptor. We identified 15 genes (out of 15,336) that were differentially expressed upon receptor-linked Aβ treatment. Genes up-regulated with Aβ treatment were associated with calcium signaling and axonal vesicle transport (including the α4 nAChR subunit, the calcineurin regulator *RCAN3*, and *KIF1C* of the kinesin family). Downregulated genes were associated with metabolic, apoptotic or DNA repair pathways (including *APBA3*, *PARP1* and *RAB11*). Validation of the differential expression was performed via qRT-PCR and immunoblot analysis in the defined model nerve cell line and primary mouse neurons. Further verification was performed using immunocytochemistry. In conclusion, we identified apparent changes in gene expression on Aβ treatment in the presence of the sensitizing nAChRs, linked to early-stage Aβ-induced neurotoxicity, which may represent novel therapeutic targets.

## Introduction

Amyloid-β (Aβ) is a short, potentially neurotoxic peptide derived from amyloid precursor protein (APP) in select regions of the brain^1,2^. At “physiological” levels (pM), there is considerable evidence for Aβ functioning as a positive neuromodulator^3–7^, acting through neuronal signaling receptors. In Alzheimer’s disease (AD), a progressive neurodegenerative disorder that is the most prevalent cause of dementia, histopathology is mainly characterized by extracellular plaques composed primarily of the Aβ peptide in fibrillar form, intracellular neurofibrillary tangles formed from hyperphosphorylated tau, and neuronal degeneration including extensive loss of cholinergic basal forebrain neurons. In addition, synaptic impairment and loss are central to changes in memory and cognition in AD^8^. Notably, during the prodromic phase of AD, soluble oligomeric Aβ levels are dramatically increased (high nM to μM) years before diagnosis (see^9^). There is ample evidence that it is the diffusible oligomeric Aβ assemblies that play a role in neurotoxicity^10^ and contribute to driving development of synaptic impairment and degeneration, largely through induction of abnormal tau and, later, neuroinflammation^11,12^. There remain important questions, however, in regard to the impact of elevated Aβ levels on neuronal function, integrity and viability, in particular altered signaling through known target receptors.

Despite extensive understanding of the pathology of AD, differential diagnosis of the disease in the prodromic and early stages has been problematic, particularly for the lack of benchmark biomarkers. Identification of novel genes linked to elevated Aβ levels during the prodromic period will contribute towards better understanding and elucidation of the mechanisms leading to neurotoxicity, and hence, neurodegeneration, and potentially provide new biomarkers for AD. While changes in Aβ levels are only correlative with stages of AD, understanding differential gene expression related to Aβ-induced toxicity pathways upon Aβ binding to known target receptors may provide new tools for study focused on Aβ neurotoxicity.

Using an *in vitro* nAChR-reconstituted nerve cell system for which we had previously established a tight timeline for Aβ-triggered toxicity, we discovered that the presence of α4β2 nAChRs, one of the notable high-affinity targets for Aβ, sensitizes the cells to toxic actions of oligomeric Aβ^13,14^, shifting the potency of Aβ for neurotoxicity from micromolar to nanomolar. We further demonstrated that this nAChR-induced Aβ neurotoxicity occurs through the timed alteration of discrete intracellular signaling molecules^14^. This prompted our study to investigate differential changes in downstream pathways underlying Aβ-linked neurotoxicity at a genetic level, possibly revealing new cellular targets for intervention in neurodegenerative processes.

The present study used two model systems. The differentiated rodent hybrid neuroblastoma NG108-15 neuronal cell line transiently expressing exogenous mouse sequences for specific nAChR subunits was employed as the defined *in vitro* nerve cell model for investigating global differential gene expression via RNA sequencing (RNA-seq) in response to sustained exposure to sensitizing levels (nM) of Aβ for neurotoxicity. Differentially regulated genes were then examined in Aβ-treated mouse hippocampal neurons as a validating primary *in vitro* neuronal model endogenously expressing nAChRs and in 5xFAD (familial Alzheimer’s disease) APP/presenilin 1 (PS1) mutant mouse hippocampus.

## Results

### Prolonged exposure of nAChR-expressing neuronal cells to soluble nanomolar Aβ differentially modulated the expression of 15 genes

**Figure 1:**
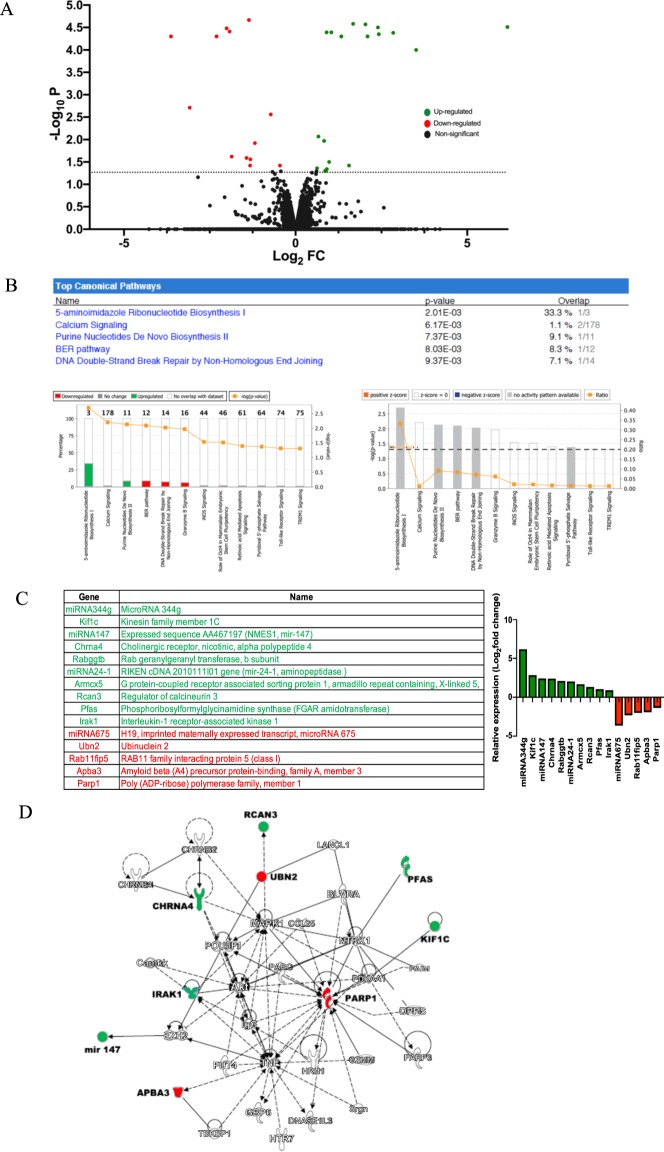
Top canonical signaling pathways and specific gene expression activated in differentiated nAChR-NG108-15 cells in response to prolonged nanomolar Aβ_1–42_ treatment as identified by deep RNA sequencing (**A**) Volcano plot (log_2_ of individual transcript fold-change (FC) as a function of the −log_10_ of p-values (P)) showing the differential gene expression of the set of 15,336 genes induced by 100 nM Aβ_1–42_ treatment in differentiated NG108-15 cells transfected with α4β2 nAChRs (nAChR-NG108-15). (**B**) Top canonical signaling pathways activated with Aβ treatment. The connecting lines (orange) indicate the ratios of genes in the identified signaling networks to total number of genes in the canonical pathways. Threshold line (right graph) indicates cut-off point of significance, *p*  <  0.05, using Fisher’s exact test for identifying particular pathways. (**C**) List of differentially regulated genes, identified as significant based on -log_10_P threshold of 2.5. The log_2_fold changes in the expression of identified genes in response to Aβ_1–42_ treatment is shown at right. (**D**) An overview of the overlapping interactions between select up-regulated or down-regulated differentially expressed genes following treatment of α4β2 nAChR-transfected cells with Aβ_1–42_ for 3 days and other intracellular signaling molecules, having a role in neurological diseases, in general. Color indicates up-regulation (green) or down-regulation (red) relative to the control cells. Solid lines represent direct interactions and dashed lines represent indirect interactions. Genes in bold are those identified via RNA sequencing. The interacting genes are: Akt, protein kinase B; Camkk; calmodulin-dependent kinase kinase; CHRN, nicotinic acetylcholine receptor; MAPK, MAP kinase; TNF, tumor necrosis factor; PARP, poly(ADP-ribose) polymerase; GBP, guanylate-binding protein; FUT, fucosyltransferase; HTR, serotonin receptor; IgG, immunoglobulin G; EZH, histone methyltransferase; HRH, histamine receptor; GZMM, granzyme; Dpp, dipeptidyl peptidase; TBKBP, TANK-binding kinase-binding protein; POU, POU homeobox; CCL, CC chemokine; BLVR, biliverdin reductase; LANCL, glutathione s-transferase; NTRK, neurotrophic tyrosine kinase receptor; PDK, pyruvate dehydrogenase kinase; FAIM, Fas apoptotic inhibitor.

As a defined *in vitro* neuronal model expressing one of the prominent receptor targets for Aβ, namely high affinity α4β2-type nicotinic receptors, which sensitize the cells to Aβ toxicity^14^, neuroblastoma hybrid rodent NG108-15 cells exclusively expressing mouse α4β2-nAChRs (nAChR-NG108-15) were treated daily with 100 nM soluble oligomeric Aβ_1–42_ as compared to vehicle-treated, receptor-expressing controls. Analysis of RNA-seq data generated from the treated cell cultures compared the levels of expression of 15,336 genes, as shown by the Volcano plot in Fig. 1A. Figure 1B lists in decreasing order of z-scores the canonical pathways activated in the nAChR-NG108-15 cells by Aβ, as identified by analysis of the RNA-seq data using the Ingenuity Pathway Analysis (IPA) tool and ranked by the highest z-scores. These canonical pathways, as ranked via IPA, included nucleotide and ribonucleotide biosynthesis, calcium signaling and DNA repair pathways including base excision repair (BER) and DNA double strand break repair by non-homologous end joining. Other activated pathways revealed on treatment with Aβ included ‘Toll-like Receptor Signaling’, ‘TREM1 Signaling’, ‘iNOS Signaling’, and ‘GranzymeB signaling’.

Of the differentially expressed genes identified via RNA sequencing, 15 were observed to be substantially altered in the neuronal cultures expressing α4β2-nAChRs on Aβ treatment as based on stringent threshold *p*-values (Fig. 1A,C). Of particular interest were *CHRNA4* (the α4 subunit of the nAChR), *KIF1C* (kinesin family), *RCAN3* (also known as *DSCR1L2*, a calcineurin regulator) and microRNA 344 g, which were up-regulated 1.3 to over 6-(log_2_)fold, and *APBA3* (X11 family, APP adapter protein also known as Mint3), *PARP1* (DNA repair family, polyADP-ribose polymerase) and microRNA 675, which were down-regulated −1.4 to −3.6-(log_2_)fold. *IRAK1* (interleukin receptor kinase), linked to Parp1 through Akt (Fig. 1D) and NFκB regulation, was only modestly changed. Regulation of nAChR, kinesin family and Rab11 family genes on Aβ treatment is consistent with previous findings with the nAChR-NG108-15 cells, where upregulation of nAChR expression and functional responses were linked to enhanced receptor recycling involving Rab11 and altered axonal mitochondrial transport^13^. The other differentially regulated genes, as identified by RNA sequencing, are novel in regard to Aβ regulation.

### Aβ-linked alteration of the expression of select genes in nAChR-expressing model neuronal cells confirmed via qRT-PCR, western blot analysis and immunocytochemistry

**Figure 2:**
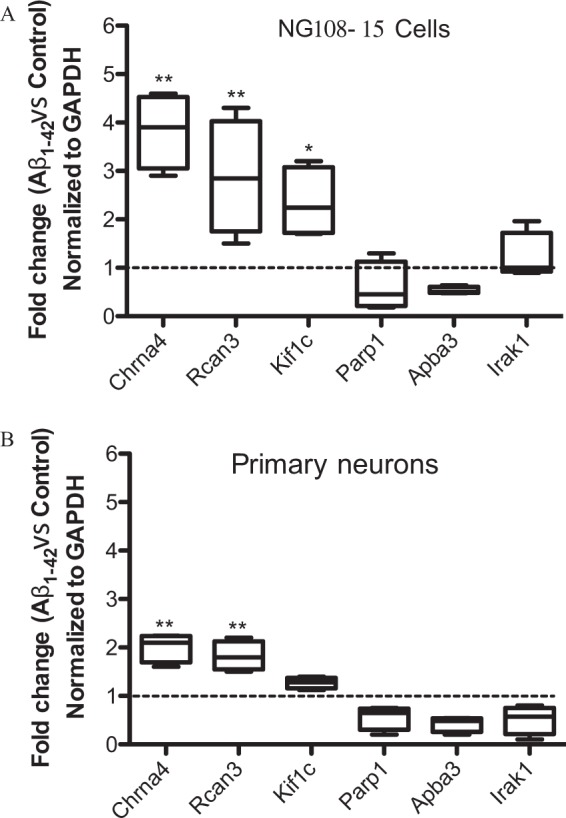
Validation of differentially expressed genes in differentiated neuroblastoma cells expressing α4β2 nAChRs treated with Aβ by qRT-PCR. (**A**) qRT-PCR was conducted on RNA extracted from α4β2 nAChR-NG018-15 cells treated or not with 100 nM Aβ_1–42_ for 3 days to determine the normalized fold-change in the gene expression of Chrna4, Rcan3, Kif1c, Parp1, Apba3 and Irak1. (**B**) The normalized fold change in the six genes in (**A**) was also validated by qPCR on RNA extracted from primary mouse hippocampal neurons treated or not with 1 µM Aβ_1–42_ for 7 days. Changes in the levels of each gene were first normalized to the GAPDH gene followed by the calculation of the fold-change in Aβ_1–42_ treated cells in comparison to control cells. Data are represented by box plots displaying the medians and 5–95% confidence intervals from four independent experiments. (*p < 0.05; **p < 0.005 relative to untreated controls).

qRT-PCR was conducted on RNA extracted from control and Aβ_1–42_-treated differentiated nAChR-NG108-15 cells to confirm the changes in Chrna4, Rcan3, Irak1, Kif1c, Apba3 and Parp1 transcripts between untreated and Aβ-treated samples, normalized with respect to GAPDH (Fig. 2A). The qRT-PCR data showed an upregulation of the levels of Chrna4, Rcan3 and Kif1c and downregulation of the Parp1, Apba1 and Irak1 transcripts, validating the RNA-seq results. A similar trend was observed for the expression levels of these transcripts in qRT-PCR conducted on RNA from primary mouse hippocampal neurons, which express endogenous nAChRs (Supplementary Fig. S2), treated or not with 1 μM Aβ_1–42_ for 7 days (Fig. 2B). (In contrast to nanomolar Aβ being sufficient for neurotoxicity in the sensitized nAChR-NG108-15 model, micromolar levels of Aβ are typically required for inducing neurotoxicity in primary neurons (e.g. see ref. ^12^)).

**Figure 3:**
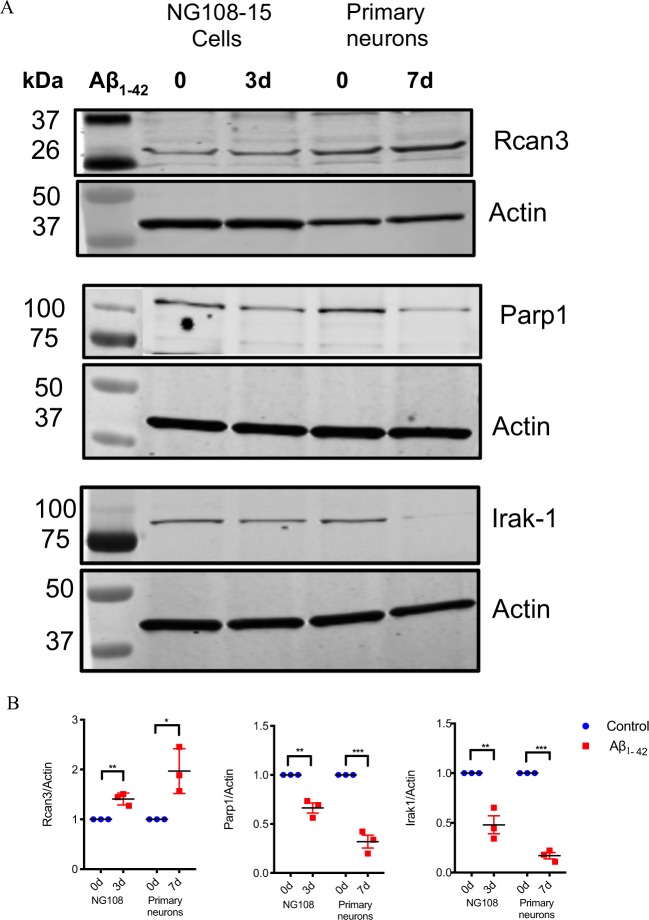
Validation of differential expression in differentiated neuroblastoma cells expressing α4β2 nAChRs or primary hippocampal neurons treated with Aβ by Western blot analysis. (**A**) Western blot analysis of Rcan3, Parp1 and Irak-1 in α4β2 nAChR-NG108-15 cells treated or not with 100 nM Aβ_1–42_ for 3 days and primary mouse hippocampal neurons treated or not with 1 µM Aβ_1–42_ for 7 days. (Supplementary Fig. S2) (**B**) The densitometric values for each protein, normalized first on the basis of the correspondent actin values to standardize the total protein loaded for each sample and then to the values at 0d (untreated). Data are presented as scatterplots displaying means ± S.D. (*p < 0.05; **p < 0.005, ***p < 0.001 relative to controls).

**Figure 4:**
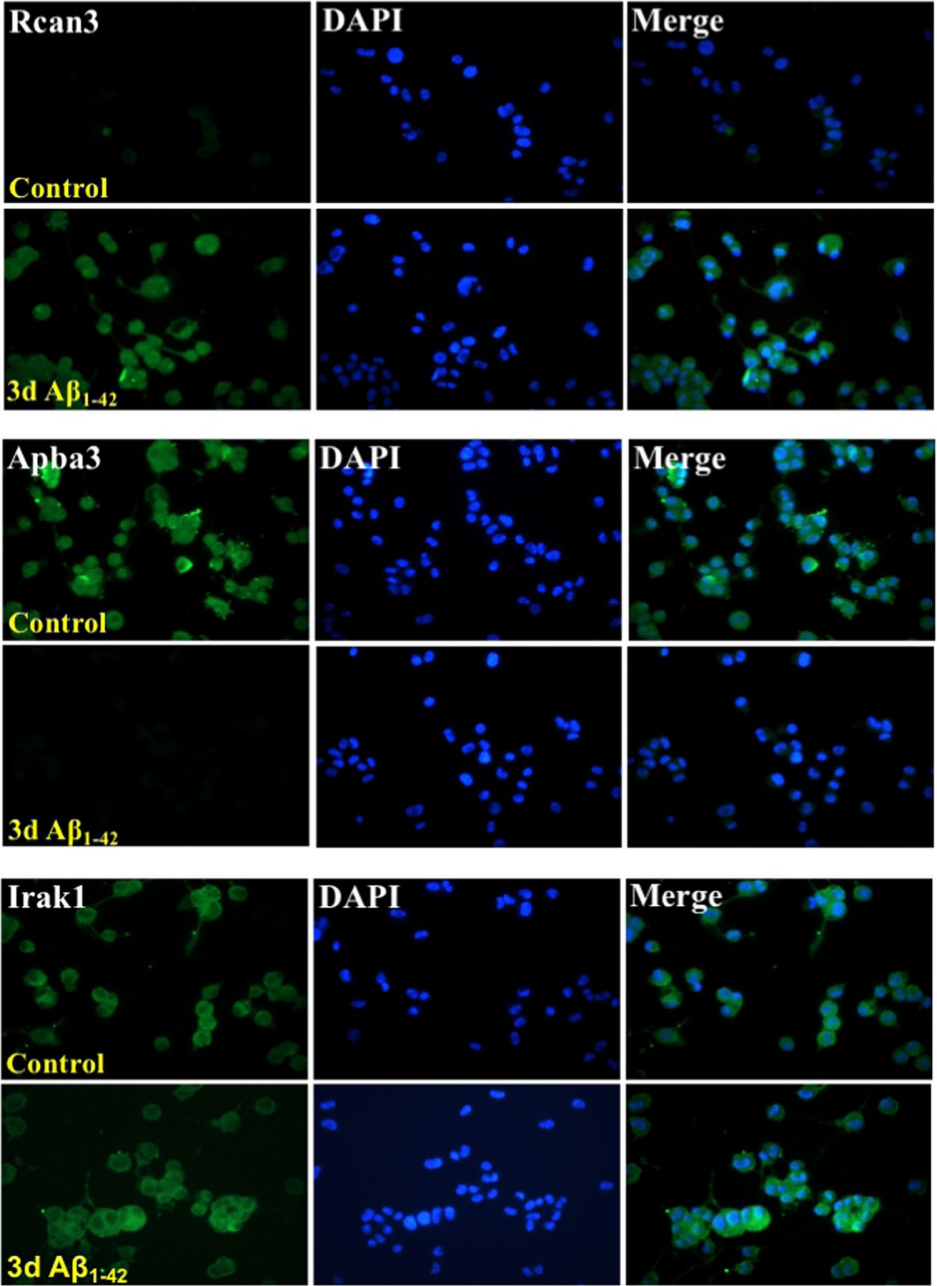
Immunostaining for Rcan3, Apba3 and Irak-1 in differentiated NG108-15 cells expressing α4β2 nAChRs and treated with Aβ. The expression levels of Rcan-3, Apba3 (Mint3), and Irak-1 were assessed by immunocytochemistry in differentiated α4β2 nAChR-NG108-15 cells treated or not with 100 nM Aβ_1–42_ for 3 days at a magnification of 20X. Nuclei were counterstained with DAPI.

The RNA-seq data were also validated at the protein expression level using immunoblot (western) analysis and immunocytochemistry. For western blot, the cell lysates collected from control and Aβ-treated neuronal cultures were used to determine changes in specific protein expression. We have previously shown an upregulation of surface levels of α4 nAChR in Aβ_1–42_-treated differentiated nAChR-NG108-15 cells^13^, which further confirms the data obtained from RNA-sequencing and also qRT-PCR. Here, we observed an up-regulation of Rcan3 and, conversely, decreased expression of Parp1 in the protein samples collected from treated nAChR-NG108-15 cells as well as treated primary hippocampal neurons (Fig. 3). The expression levels of Irak1 were modestly altered in the nAChR-NG108-15 cells and reduced in the hippocampal neurons (Fig. 4). Immunostaining for Rcan3, Apba3 and Irak1 (Fig. 4) of control and Aβ-treated differentiated NG108-15 cells further affirmed these results. The difference between means of Aβ-treated and control cells was 25.40 ± 3.962 (p < 0.05) and −1.438 ± 2.021 (p > 0.05) for Rcan3 and Irak1, respectively.

**Figure 5:**
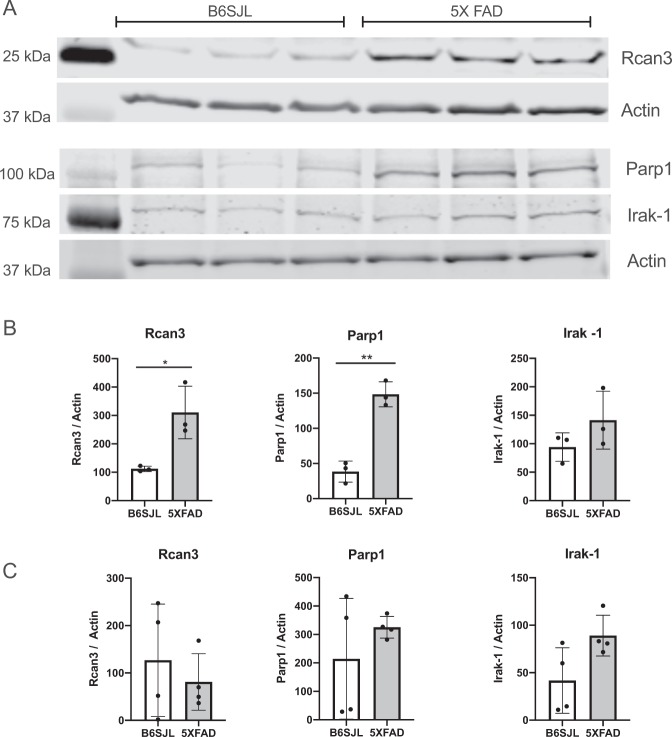
Validation of differential expression in 1.5-month-old B6SJL and 5XFAD mouse model hippocampal lysates. (**A**) Western blot analysis of 1.5-month-old B6SJL (*n* = 3) and 5XFAD (*n* = 3) mouse hippocampal lysates. M_r_ for Rcan3, Parp-1, and Irak-1 are 26 kDa, 113 kDa, and 80 kDa, respectively. (Supplementary Fig. S3) (**B**) The densitometric values for each protein normalized to corresponding loading control (actin) for each sample. Corresponding *p*-values for Rcan3 and Parp-1 are 0.02 and 0.0012, respectively. Data are presented as means ± S.D. (*p < 0.05; **p < 0.005; ***p < 0.001). (**C**) The densitometric values for each protein from 8–8.5 month-old B6SJL and 5XFAD mouse hippocampal lysates normalized to corresponding loading control (Actin) for each sample (*n* = 4). (Supplementary Fig. S4) Data are presented as means ± S.D.

Changes in expression of these representative proteins in the hippocampi from a familiar AD mouse model, 5xFAD (APP/PS1 mutant mice), at 1.5 months of age when Aβ levels begin to rise (see^15^), as compared to age-matched background control mice, were also observed, particularly upregulation of Rcan3 (Fig. 5), which has a predominant neuronal localization^16^. Parp1 was also upregulated; however, it is expressed across a wide range of cell types but is most predominantly expressed in glia^16^, and thus the change in expression may have been significantly affected by non-neuronal cells. There was no significant difference in the expression of Irak1. These differences were not observed in 8–8.5-month-old 5xFAD hippocampal extracts, an age when the mice display robust AD-like endophenotypes of synaptic dysfunction and spatial memory deficits.

### Analysis of pathways modulated by Aβ_1–42_ treatment in the presence of α4β2 nAChRs

**Figure 6:**
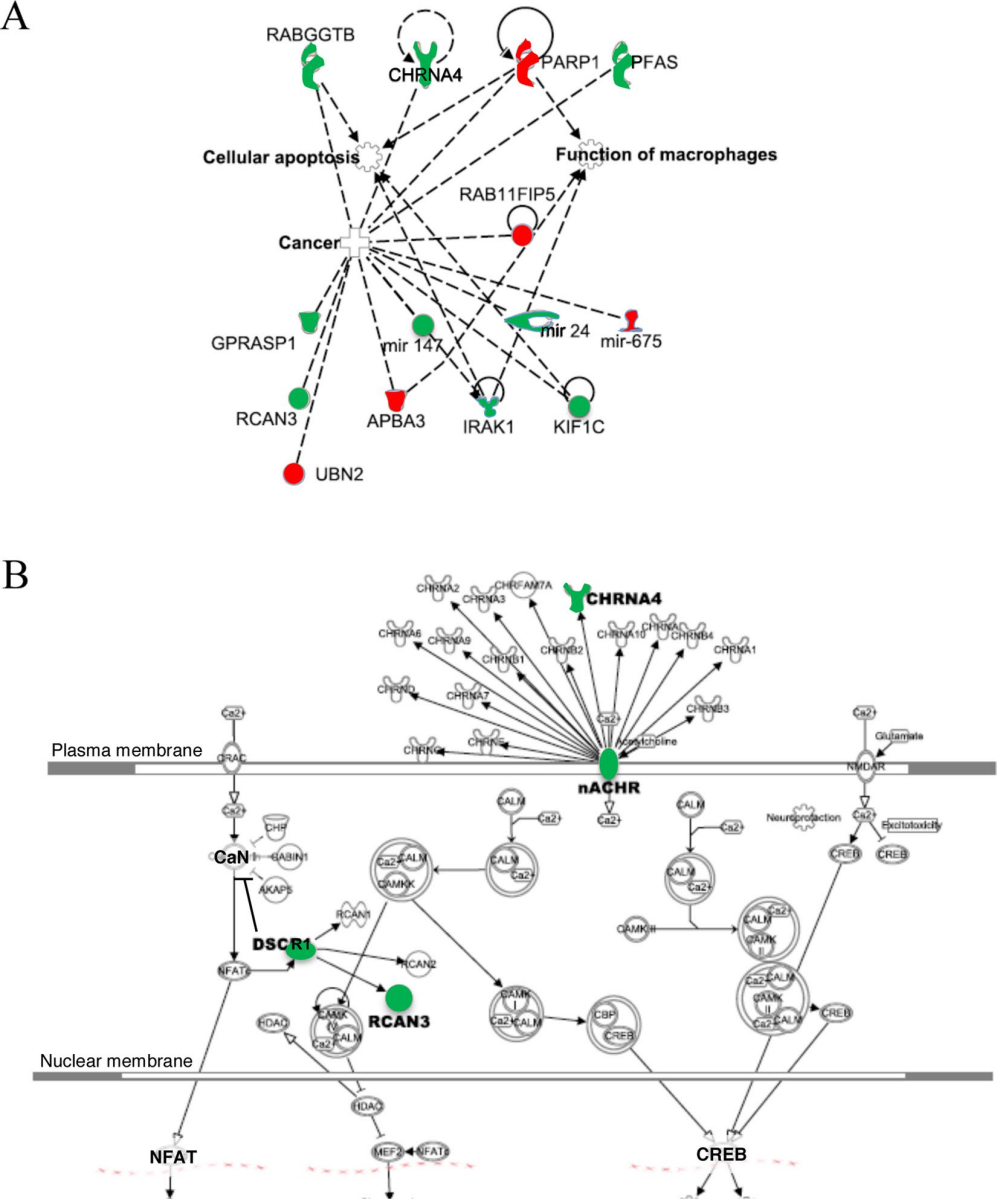
Functional connections among the differentially expressed genes following prolonged Aβ_1–42_ treatment of differentiated NG108-15 cells expressing α4β2 nAChRs, including calcium signaling. (**A**) Functional connections between top ranked 15 up-regulated or down-regulated differentially expressed genes following treatment of α4β2 nAChR-NG108-15 cells with Aβ_1–42_ for 3 days identified via IPA. Color intensity indicates the degree of up-regulation (green) or down-regulation (red) relative to the control cells. Dashed lines represent connections to function or pathology. (**B**) Genes for nAChR subunits (CHRNAs) are shown at the top (plasma membrane). Intracellular calcium signaling pathways, including links to Rcans, are as shown. Pathways were identified by IPA. Abbreviations: CALM, calmodulin; CaN, calcineurin; CAMK, calmodulin-dependent protein kinases; NFAT, nuclear factor of activated T-cells; HDAC, histone deacetylase; CREB, cAMP response element binding protein; CBP, CREB binding protein; NMDAR, NMDA-type glutamate receptor; DSCRs, Rcans (regulators of calcineurin).

To investigate the biological interactions of differentially expressed genes and identify functional networks, the prominent genes differentially expressed in response to Aβ treatment that were identified in the RNA-seq analysis were investigated using IPA, as previously described. Figure 1D shows the interactions between various genes, notably those involved in apoptosis and DNA repair pathways. One of the characteristic hallmarks of AD pathogenesis is an increase in oxidative damage to DNA. We have previously shown in our *in vitro* nAChR-NG108-15 neurotoxicity model that prolonged exposure to Aβ causes increased oxidative stress in a manner dependent upon the presence of the sensitizing α4β2-nAChRs^14^, leading to apoptosis. The gene network analysis of the differentially regulated genes identified here indicates that the Aβ-triggered increase in oxidative DNA damage and apoptosis (Fig. 6A) is correlated, in part, with a defective BER pathway (Fig. 1A), in line with ample evidence showing that Aβ can have deleterious effects on DNA repair pathways including downregulation of *BER*-associated genes^17^. In addition, Parp1 (poly(ADP-ribosyl) polymerase 1), a DNA repair enzyme that catalyzes the formation of poly ADP-ribose polymers from nicotinamide adenine dinucleotide (NAD^+^), is usually activated by single-strand breaks associated with oxidative stress, and in the present study was found to be downregulated in neuronal cultures (Figs. 1–3) but upregulated in APP/PS1 5XFAD mouse hippocampal lysates (Fig. 5), which contain a mixture of neuronal and non-neuronal proteins.

One of the other prominent pathways that emerged in this study is calcium signaling (Figs. 1B and 6B). We have previously shown that Aβ induces changes in Ca^2+^ levels via exogenous α7-nAChRs or α4β2-nAChRs expressed in the somata and axonal varicosities of differentiated NG108-15 cells^14,18,19^, as an early event in the Aβ toxicity timeline. The data from RNA sequencing highlighted a critical molecule involved in calcium signaling, notably Rcan3, a calcineurin regulator. The specific role of Rcan in Aβ neurotoxicity remains to be determined.

## Discussion

A direct agonist-like action of soluble picomolar-nanomolar Aβ via nAChRs in the regulation of presynaptic calcium was previously observed^6,18,19^. The presence of α4β2-nAChRs, in particular, was found to significantly sensitize the cells to chronic Aβ-induced oxidative stress and, ultimately, apoptosis^13^. In order to elucidate the pathways involved in chronic Aβ-induced toxicity linked to a defined Aβ target, we attempted to determine the differential gene expression on prolonged treatment with nanomolar Aβ in the presence of sensitizing nAChRs through a transcriptome profile. Our study is the first of its kind to use a defined *in vitro* neuronal toxicity model reconstituted with high-affinity targets for Aβ conferring sensitization for the initiation of Aβ neurotoxicity. In this study, we discovered that prolonged exposure of a model nerve cell line exclusively expressing α4β2 nAChRs (nAChR-NG108-15 cells) to nanomolar Aβ resulted in substantial alteration in the expression of a unique set of 15 genes, confirmed first by quantitative transcript analysis (qPCR) and then, for prominent examples, protein analysis (immunoblot and immunostaining). Most of the 15 differentially expressed genes are distinct from genes differentially expressed in a different neuroblastoma model subjected to short-term treatment with micromolar levels of an Aβ toxic fragment (Aβ_25–35_) using microarray analysis^20^. However, many of the canonical pathways noted in the present study, such as those involved in preapoptotic or apoptotic processes, were also prominent in the latter study^20^. The genes and pathways identified here in our acute Aβ neurotoxicity models are also in contrast to genes strongly correlated with late-onset AD, including apolipoprotein E (*APOE*), triggering receptor expressed on myeloid cells 2 (*TREM2*) and cluster of differentiation 33 (*CD33*)^21–24^ among others, suggesting differences in gene regulation with early rises in Aβ in brain (prodromic period) as compared to AD.

Out of several genes displaying differential expression in the model nerve cells expressing α4β2-nAChRs on treatment with nanomolar Aβ, upregulation of *CHRNA4* and *RCAN3* is of particular interest. While upregulation of high-affinity Aβ target nAChRs with prolonged Aβ was previously described, regulation of *RCAN* by Aβ is a novel observation. Rcans (regulators of calcineurin^25^), also previously known as calcipressins as well as Down Syndrome Critical Region‐1 (DSCR1)‐like proteins, constitute a conserved family of proteins from yeast to humans and bind calcineurin to modulate its activity^26,27^. Studies have shown that the transcripts for all mammalian Rcans (Rcan1, Rcan2, Rcan3) are expressed in the brain^28,29^. The critical role that Rcan proteins play in the physiology of brain is highlighted in reports showing increased locomotor activity and impaired working memory in Rcan1/Rcan2 double‐knockout mice^30^. Interestingly, some studies show that there is a link between oxidative stress-induced Rcan levels and aging (and AD-related pathology). Specifically, Cook and colleagues^31^ demonstrated that Rcan protein expression was upregulated in the pyramidal neurons of the temporal lobe with aging. It was further shown that there was a positive correlation between the total number of calcipressin (Rcan)-positive pyramidal neurons and the number of neurofibrillary tangles in the temporal cortex.

Among the other genes altered in response to treatment with Aβ in the presence of nAChRs was Parp1, where, interestingly, down-regulation in our *in vitro* neuronal culture model as well as primary mouse hippocampal neurons was observed, while upregulation was evident in APP/PS1 (5XFAD) mouse hippocampus at 1.5 month of age when Aβ levels first rise significantly^15^, consistent with increased Parp1 activity found in hippocampus and entorhinal cortex of TgCRND8 (double mutant APP at KM670/671NL + V717F) mice at an equivalent stage (3 months)^32^. A prominent role for Parp1 in carrying out polyADP-ribosylation (up to 93% in the brain) and hence, maintaining DNA integrity has been described^33^. However, there is now ample evidence indicating that Parp1 also plays a significant role in cell death processes as well as regulation of mitochondrial function^34^. We have also previously shown a disruption in mitochondrial function and transport along axons in our *in vitro* model nerve cell culture system (nAChR-NG10815) in response to Aβ treatment^13^. There are also reports suggesting that Parp1 may play a crucial role in the possible interactions between molecules involved in AD-related pathology and regulation of mitochondrial function^35^. As Parp1 has been found to have predominantly glial expression (see 16), a putative differential regulation of Parp1 in neurons vs. glia by Aβ, inferred from our findings, may indicate, in turn, a differential regulation of mitochondrial function in different cell types in brain. Thus, the impact of Aβ in brain on Parp1 activity should be revisited for neuron vs. glia in susceptible brain regions.

Yet another class of RNA that showed novel changes in differential expression on exposure to Aβ in the presence of sensitizing nAChRs were microRNAs (or miRNAs). MicroRNAs, which are short (~21–23 nucleotides) conserved non-protein-coding RNAs transcribed from the genome^36^, constitute a very important class of regulators of gene expression^37,38^. Our data showed that the microRNA miR344g, whose role in the nervous system is yet to be determined, was the most strongly upregulated transcript of all those detected with RNA-seq on treatment of nAChR-NG108-15 cells with nanomolar Aβ. In addition, several other microRNAs including miR147, linked to Toll-like receptors, and miR24-1, linked to enhancer RNA expression, were among the strongly upregulated transcripts found on exposure of the nAChR-NG108-15 cultures to Aβ. In contrast, the microRNA miR675, a regulator of cell proliferation, was significantly downregulated on treatment with Aβ. At present, the possible roles for these various microRNAs in Aβ neurotoxicity remain to be discovered, as are the ramifications of these findings for the prodromic period of AD.

In sum, our transcriptome profile of model neuronal systems expressing high-affinity, sensitizing target nAChRs exposed to prolonged treatment with Aβ revealed novel gene regulation, including two notable genes, *RCAN* and *PARP1*, involved in the regulation of two key early events in Aβ neurotoxicity, namely calcium signaling and mitochondrial function, respectively. The differential expression of these two genes could thus serve as novel biomarkers for Aβ toxicity, perhaps in the prodromic stage prior to AD, to be confirmed in AD models and patients. Furthermore, characterizing the genes associated with neuronal dysfunction and death after Aβ treatment will have significant impact on developing neuroprotective agents to reduce or prevent pathogenesis leading to AD.

## Materials and Methods

### Nerve cell culture and transfection

Differentiated hybrid neuroblastoma NG108–15 cells were used as a defined model nerve cell system, as they are normally devoid of functional nAChRs. The cells were maintained in Dulbecco’s modified Eagle’s medium (DMEM) containing 15% FBS and hypoxanthine/ aminopterin/thymidine (HAT selection). Cells were differentiated on poly-L-lysine (plates) or Cell-Tak (coverslips) with 1 mM dibutyryl cyclic AMP in DMEM in the presence of reduced serum (1% FBS) and penicillin-streptomycin-glutamine for 72 h, as described previously^13^. pcDNA3.1 expression vectors harboring mouse sequences for α4- and β2- nAChR subunits were transfected at 1:4 ratio, respectively, into the differentiated cells using FuGENE HD (ThermoFisher), a lipid-based transfection reagent, and the cultures were incubated for 48 h. The transfected cells were then treated or not (control), with 100 nM (sensitizing concentration) of Aβ_1–42_ for 3 days, the time period based on the established timeframe for Aβ-induced toxicity in this nerve cell model system^13,14^. The Aβ treatment was therefore initiated following nerve cell differentiation and was listed as 0–3 days.

### Primary hippocampal culture

Primary hippocampal neuron cultures were prepared from neonatal (1–2d old) C57/B6J mouse pups^39^ under an approved University of Hawaii IACUC protocol (16-2282-3) in accordance to all guidelines and regulations. Following rapid decapitation, brains were removed from the mice into ice‐cold Neurobasal A medium (NB) containing B‐27 supplement, 5% fetal bovine serum and Gentamicin (Serum NB). Hippocampi were then dissected out under a stereomicroscope. The hippocampi were digested with activated papain (Worthington) in Hanks buffer with 10 mM cysteine at 37 °C for 15 mins. The preparations were washed by centrifugation in Serum NB. The cells were dissociated using sequential trituration with fire-polished Pasteur pipettes of decreasing diameter and collected by low‐speed centrifugation. The dissociated cells were pre‐plated in standard tissue culture dishes to remove adherent non‐neuronal cells (glia; fibroblasts) for 10–15 min. The neuron‐enriched supernatant was diluted to 1 × 10^5^ cells/mL and plated into poly‐*D*‐lysine‐coated 6‐well plates in Serum NB. The cultures were maintained in Neurobasal A medium containing B-27 and Gentamicin for 7 days to select for neurons^14^, followed by treatment with 1 µM Aβ_1–42_ for another 7 days, the treatment time period based on the observed timeframe for toxicity^39^. The Aβ treatment was therefore initiated following establishment of neuronal cultures and was listed as 0–7 days.

### Animals

Animal husbandry and euthanasia were performed under an approved Institutional Animal Care and Use (IACUC) protocol (11-1219-6/16-2282-2) in conjunction with NIH guidelines for use of vertebrate animals in research. The transgenic mutant APP/PS1 mouse line, 5×FAD (familial AD) on the B6.SJL background (B6SJL-Tg (APPSwFlLOn, PS1 (PSEN1)*M146L*L286V) 6799Vas/Mmjax originally obtained from JAX stock #006554, MMRRC034840 hemizygous), was used as a well characterized model for Aβ-based pathology and neurodegeneration^15^, along with age-matched control (B6.SJL background) mice (MMRRC034840 Non-carrier). Age-matched mice (either sex) from the 5XFAD and B6.SJL colonies were housed in ventilated enrichment cages in the John A. Burns School of Medicine AAALAC-accredited Vivarium with *ad libitum* access to food and water. Mice were used at 1.5 months of age and 8–8.5 months of age.

### Aβ preparation

Soluble solutions of Aβ_1–42_ (American Peptide; Anaspec) were prepared from aqueous stock solutions, followed by brief bath sonication. This Aβ preparation was previously shown to exist predominantly in the oligomeric state^19,40^.

### RNA-seq sample preparation and gene expression analysis

Total RNA was isolated from the cells using PureLink® RNA Mini Kit (Ambion, Life Technologies, #12183025) as per the manufacturer’s protocol. Genomic DNA contamination was eliminated from the RNA preparation by digesting with RNase-free DNase (Qiagen). The quality of all RNA samples was determined using an Agilent Bioanalyzer 2100 and the precise quantity determined via Qubit (National Center for Genomic Resources, New Mexico). Multiplexed RNA-seq libraries were prepared from the cellular RNA and paired-end 100-bp sequencing was conducted using an Illumina HiSeq. 2500 sequencer (National Center for Genomic Resources, New Mexico).

A total of 146,247,335 reads were inspected using the FastQC program (http://www.bioinformatics.babraham.ac.uk/projects/fastqc) and minimally trimmed using Trimmomatic (v. 34)^41^ to remove low-quality bases. The processed reads were then mapped to the mouse genome (ref mm10) using TopHat (v.2.1.1). The programs Cufflinks and Cuffdiff (v.2.2.1)^42^ of the Tuxedo suite were subsequently used to assemble transcripts and to assess statistically significant differential expression changes between the control and treated groups, based on an FDR-adjusted *p*-value of 0.05.

### Pathway analysis

The RNA-seq data were analyzed using the Ingenuity Pathway Analysis (IPA, Qiagen). Canonical pathways and functional processes of biological importance were assessed using the list of differentially expressed genes identified by RNA-seq and the IPA Knowledge Base, as described previously^43^. Pathway enrichment *p*-values (Fisher’s exact test) and activation *z*-scores were calculated by IPA. The significance threshold was set at p  <  0.05 as the cut-off. For positive z-scores, pathways at or above the threshold (expressed as −log p values) were delineated by the IPA software as a ranked list.

### Validation by qRT-PCR

qRT-PCR was conducted on RNA samples from control and Aβ-treated cells. Total RNA was extracted as described in the previous section. The iScript™ cDNA Synthesis Kit (Bio-Rad) was used to synthesize cDNA. The mRNA levels of various genes were determined using qRT-PCR (Applied Biosystems™ 7500) and the fold-changes in Aβ-treated samples compared to untreated ones were calculated after normalizing to the GAPDH gene expression. The primer sequences used for qRT-PCR are listed in Table 1.

**Table 1:** Primer sequences used for qRT-PCR.

Gene (Accession number)	Sequence (5′-3′)
***Chrna4*** (NM_015730.5)	
Forward	ATGTCAGGAAGGAGGTAT
Reverse	CAATATCCAGAGTTCAGAGA
***Rcan3*** (NM_022980.4)	
Forward	TTGGTTGGTTGGTTGATT
Reverse	AAGGAGGAAGCATAGACT
***Kif1c*** (NM_153103.2)	
Forward	CTACTGGCTACCTTGATT
Reverse	TTCTTGCTTACACTTATTCTC
***Parp1*** (NM_007415.3)	
Forward	TACCATCCAACTTGCTTT
Reverse	CTTCATCTGTTCCATCCA
***Apba3*** (NM_018758.2)	
Forward	CGTTTGAGAGTGTGTATG
Reverse	CTACAGGTGACAGATTCC
***Irak1*** (NM_001177973.1)	
Forward	CTTGGATTTAGAACCTGAAA
Reverse	GCACACTATGAGAACTTC

### Validation by Western immunoblot analysis

Protein samples were extracted from various cell cultures or mouse hippocampi isolated from 1.5- month-old 5XFAD, 8–8.5-month-old 5XFAD or B6.SJL 9 (control) mice using 1% SDS as lysis buffer, followed by sonication for 10 min (cells) or needle homogenization (hippocampi) and centrifugation at >12,000 rpm for 20 min at 4 °C. The total amount of protein was quantified by a Pierce™ BCA Protein Assay Kit (ThermoFisher Scientific, # 23225). The SDS-solubilized protein samples were diluted into sample gel buffer containing reducing agent, boiled at 95 °C for 10 min, immediately cooled on ice and then centrifuged. Equal amounts of protein were subjected to electrophoretic separation on 4–20% gradient Tris-Glycine polyacrylamide gels (Bio-Rad or ThermoFisher). The proteins in the gels were transferred onto either PVDF membrane (cell extracts) or nitrocellulose (hippocampal extracts) using an iBlot2 semi-dry blot transfer system (ThermoScientific). The blots were treated with LI-COR Odyssey Blocking buffer and then incubated with affinity-purified rabbit anti-Rcan3 (calcipressin3; RRID:AB_2179558), anti-Parp1 (RRID:AB_11000218) or anti-Irak1 (RRID:AB_2532270) primary antibodies (typically at 1:1000; ThermoFisher) and anti-actin monoclonal antibody (RRID:AB_476697; Sigma-Aldrich,) overnight at 4 °C. The transfer blots were washed 3× (10 min each wash) in 0.1% Tween-20 in Tris-buffered saline and incubated with the appropriate IR-dye-conjugated secondary antibody (anti-rabbit and/or anti-mouse; 1:5000; LI-COR Biosciences) for 1 h. An Odyssey IR imaging system was used for signal detection. Analysis was performed via Image Studio v5.2.5 software (LI-COR Biosciences). Immunoreactivity for actin was used as a loading control for all samples.

### Validation by immunocytochemistry

Cell cultures were fixed with freshly prepared 4% paraformaldehyde in HBS at room temperature for 40 min and rinsed with phosphate-buffered saline (PBS) for 30 min. The cultures were then permeabilized using 0.1% Triton-X in Tris-buffered saline (TBS) followed by washing with TBS for 20 min. Thereafter, a blocking buffer containing 5% bovine serum albumin and 10% normal goat serum in TBS was added to the cells for 30 min to block nonspecific binding. Affinity-purified primary antibodies (anti-Rcan3 (calcipressin3), anti-Apba3 (RRID:AB_2057069) and anti-Irak1; at 1:100; Fisher Scientific) were then added to the cultures and incubated overnight at 4 °C. The cultures were washed with 10% goat serum in TBS for 30 min, and incubated with the FITC-conjugated secondary IgG antibodies (typically at 1:500) and DAPI to label cell nuclei for 30 min at room temperature. The coverslips were finally washed with 10% normal goat serum and TBS and plated onto glass microscope slides, and sealed in Vectashield anti-fade mounting media (Vector Laboratories). The immunostained preparations were subsequently visualized using an Olympus IX71 fluorescence microscope with appropriate fluorescence filters via a 20X objective and images captured via CCD camera. Digitized images were analyzed via ImageJ.

### Statistical analysis

Samples, as biological replicates, were from independent cultures (for each condition: RNA-seq, *n* = 4; qRT-PCR, *n* = 3; immunoblot, *n* = 3). Differentially expressed genes (treated vs. untreated) were assessed as significant based on an FDR (false discovery rate)-adjusted *p*-value (q-value) of <0.05 under the Cuffdiff test statistics (Cufflinks)^39^. For qRT-PCR and immunoblot analysis, Student *t*-tests (treated vs. untreated) were performed using Graphpad Prism (v.8) following testing for data normality, with *p*  <  0.05 the minimum value for significance (as rejection of the null hypothesis).

## Supplementary information

Supplementary Figures.

## Data Availability

Datasets generated on analysis of the RNA-sequencing are available from the corresponding author on reasonable request. The model neuronal cell line used, NG108-15, is readily available from the American Type Culture Collection.
