# Supplementary material for: Transcriptome Profile of Nicotinic Receptor-Linked Sensitization of Beta Amyloid Neurotoxicity
Source: Sci Rep. 2020 Mar 30;10:5696. doi: 10.1038/s41598-020-62726-0 (PMC7105468; doi:10.1038/s41598-020-62726-0)
Supplement: Supplementary file 1 — Supplementary Figures. [file 41598_2020_62726_MOESM1_ESM.docx]

**Transcriptome Profile of Nicotinic Receptor-Linked Sensitization**

**of Beta Amyloid Neurotoxicity**

Komal Arora^1^*^#^*, Mahdi Belcaid^2¶^, Megan J. Lantz^1^, Ruth Taketa^1^ and Robert A. Nichols^1*^

*From the ^1^Department of Cell and Molecular Biology, John A. Burns School of Medicine, University of Hawai’i; ^2^Pacific Center for Emerging Infectious Diseases Research, John A. Burns School of Medicine, University of Hawai’i*

Running title: Differential gene expression on Aβ-induced neurotoxicity through nAChRs

**Supplementary Information**

**Immunostaining for nAChRs in the primary neuron cultures**

**
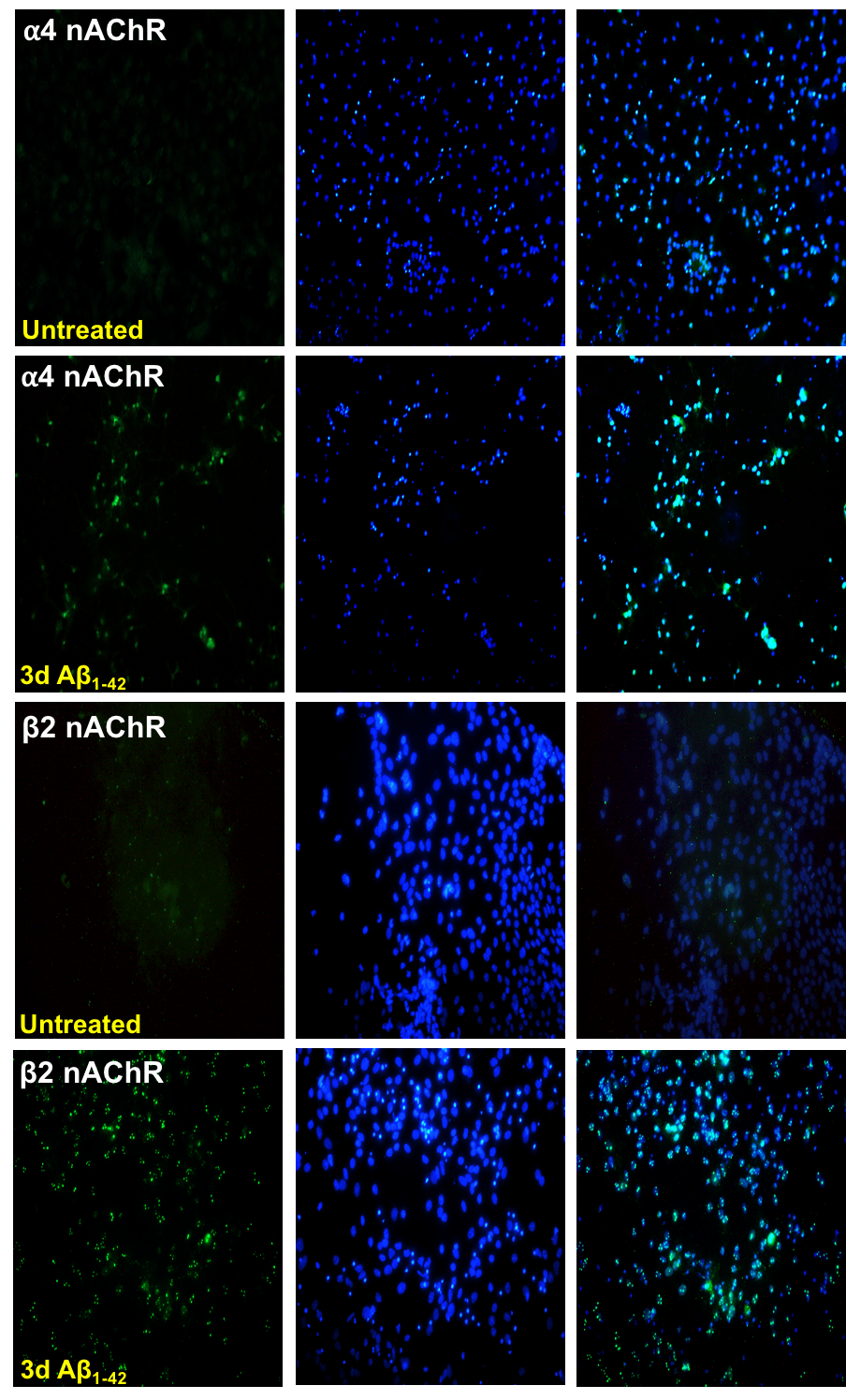
**

**Supplementary Figure S1**

Mouse hippocampal neuron cultures were treated with Aβ or not (untreated control) for 3d, fixed and then immunostained for the α4 or β2 subunits of the α4β2-nAChR (green: 1^st^ column). Immuno-stained cultures were counterstained with DAPI to label all cells (blue: 2^nd^ column). Merged images are presented in the 3^rd^ column.

**Immunoblots of Rcan3, Parp1 and Irak1 expression in response to Aβ in NG108-15 cells and primary hippocampal neurons**


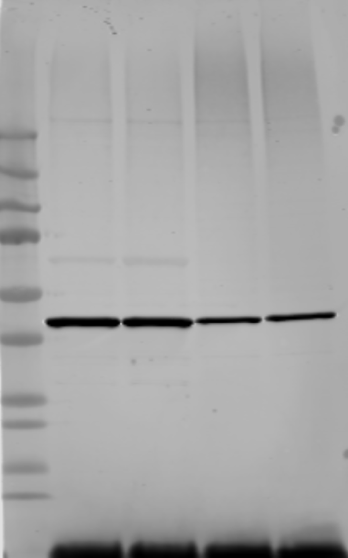

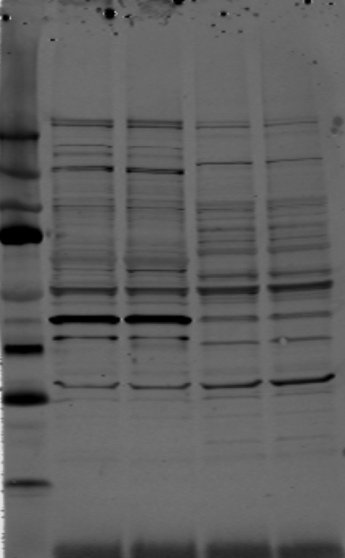
 Rcan3 Actin

**Supplementary Figure S2**

Full-length immunoblots (left: 800nm channel) for the representative cropped blots in Figure 3A, showing the markers (right) with the accompanying actin immunolabeling in the second channel (right: 700nm) imaged on the same blots (dual-channel LI-COR Odyssey gel imager). Bands of interest were identified by Mw.

Parp1 Actin


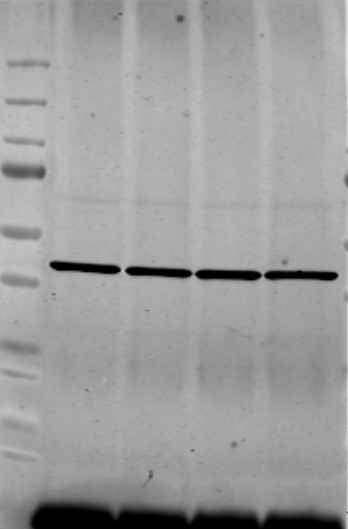

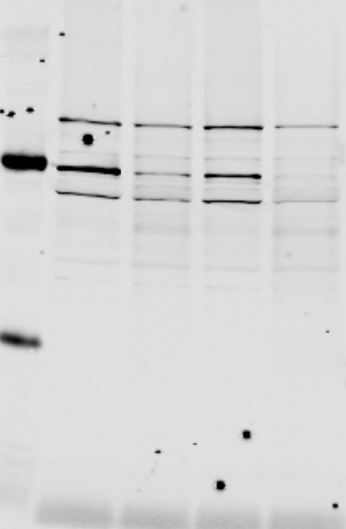


Irak1 Actin


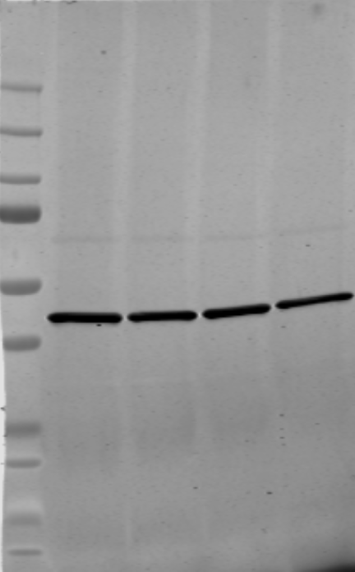

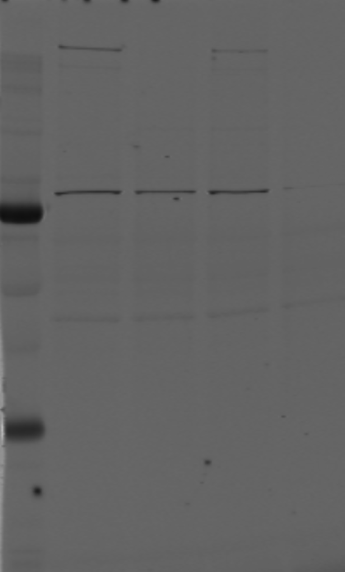


**Immunoblots of Rcan3, Parp1 and Irak1 in hippocampal lysates from 1.5-month-old B6SJL and 5X FAD mice**

**Supplementary Figure S3**

Full immunoblots of hippocampal lysates from 1.5-month-old B6SJL and 5X FAD mice. Each blot represents the same set of samples for B6SJL and 5XFAD hippocampal lysates (*n*=3 each). The protein of interest was normalized to the corresponding loading control (Actin) for each sample. (**A**) Full immunoblot probed for Rcan3 (27 kDa), Irak-1 (80 kDa), and loading control, Actin (42 kDa). (**B**) Full immunoblot probed for Parp1 (113 kDa) and loading control, Actin (42 kDa).

**Immunoblots of Rcan3, Parp1 and Irak1 in hippocampal lysates from 8-8.5-month-old B6SJL and 5X FAD mice**


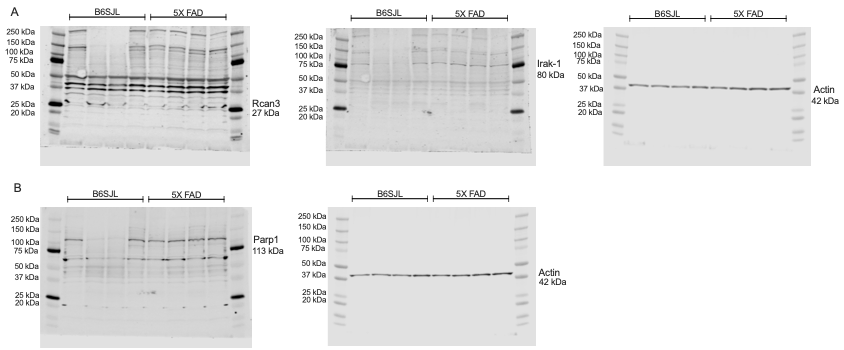


**Supplementary Figure S4**

Full immunoblots of hippocampal lysates from 8-8.5 month-old B6SJL and 5X FAD mice. Each blot represents the same set of samples for B6SJL and 5XFAD hippocampal lysates (*n*=4 each). The protein of interest was normalized to the corresponding loading control (Actin) for each sample. (**A**) Full immunoblot probed for Rcan3 (27 kDa), Irak-1 (80 kDa), and loading control, Actin (42 kDa). (**B**) Full immunoblot probed for Parp1 (113 kDa) and loading control, Actin (42 kDa).
